# Effects of COVID-19 lockdowns on unintended pregnancies among adolescent girls and young women in low- and middle-income countries: a scoping review

**DOI:** 10.1186/s12978-025-02045-7

**Published:** 2025-05-22

**Authors:** Lara Teresa Lüdecke, Björn Ekman, Malachi Ochieng Arunda, Robert Bulamba, Alex Daama, Anna Mia Ekström, Emmanuel Kyasanku, Elin C. Larsson, James Nkale, Jesper Sundewall

**Affiliations:** 1 Social Medicine and Global Health, Department of Clinical Sciences, Lund University, Malmö, Sweden; 2 Department of Global Public Health, Karolinska Institutet, Stockholm, Sweden; 3 Africa Medical and Behavioural Sciences Organization (AMBSO), Wakiso, Uganda; 4 Department of Infectious Diseases/Venhälsan, South General Hospital, Stockholm, Sweden; 5 WHO Collaborating Centre for Research and Research Training in Human Reproduction, Department of Women’s and Children’s Health, Karolinska Institutet, Stockholm, Sweden; 6 HEARD, University of KwaZulu-Natal, Durban, South Africa

**Keywords:** Unintended pregnancies, Adolescent girls, Young women, COVID-19, Lockdown, Sexual and reproductive health and rights, Low- and middle-income countries, School closures

## Abstract

**Background:**

The response to the COVID-19 pandemic involved various lockdown measures, including school closures, which significantly impacted young populations, particularly in low-and middle-income countries (LMICs). Given the well-known protective effect of regular school attendance on sexual and reproductive health and rights (SRHR), reports of disrupted education, compromised SRHR, and an increase in unintended pregnancies among adolescent girls and young women (AGYW 10–24 years) have caused major concerns. We conducted a scoping review to compile the available evidence of the impact of COVID-19 lockdown measures on unintended pregnancies among AGYW aged 10–24 in LMICs.

**Methods:**

The scoping review followed the five-stage framework by Arksey and O´Malley. A systematic search on two comprehensive databases, using search terms related to COVID-19 and unintended pregnancies, was conducted along with grey literature searches of articles in English language published between 2019 and 2024. The systematic review software Covidence was used for publication screening, selection and data extraction.

**Results:**

After deduplication, 241 publications were screened, and 72 full-text publications were assessed for eligibility. 13 publications from the database searches, citation searching and grey literature, were included. The scoping review included seven studies that applied quantitative methods, four that used qualitative approaches and the remaining two mixed methods. Five out of 13 publications were set in Uganda, two in Kenya and two in Nigeria, while six other countries were represented once. All included studies reported increases in pregnancies among AGYW during the COVID-19 period. School dropout among girls following a pregnancy was reported to have increased. Factors associated with unintended pregnancy were school closures, limited access to SRHR services including contraceptives, and increasing sexual encounters.

**Conclusion:**

School closures and other lockdown measures during the COVID-19 pandemic led to an increase in unintended pregnancies among AGYW in LMICs. The long-term consequences for these young individuals, their communities and to the broader society are still to be measured and available evidence is limited, few studies have applied robust study designs, and several relied on small sample sizes. Further research is needed to build a stronger evidence base for health and socio-economic impacts of school closures and lockdowns among young people.

**Supplementary Information:**

The online version contains supplementary material available at 10.1186/s12978-025-02045-7.

## Plain Language Summary

The contagiousness of novel coronavirus was accelerating globally (China reported the first occurrence in December 2019). As a result, a Public Health Emergency of International Concern was announced by the World Health Organization (WHO) on January 30, 2020 and subsequently, a pandemic was declared in March 2020. The pandemic led to various lockdowns, including school closures, significantly affecting young people, especially in low-and middle-income countries (LMICs). Schools are important for protecting the sexual and reproductive health of adolescents. When schools closed and access to health services was limited, concerns about more unintended pregnancies among girls and young women aged 10-24 increased.

We conducted a review to gather already existing evidence on this issue. We searched databases and websites of key international organizations for articles published between 2019 and 2024. In total, 13 articles met our inclusion criteria.

Our review found that all studies reported an increase in pregnancies among adolescent girls and young women during the COVID-19 period. There was also an increase in girls dropping out of school after becoming pregnant. The main reasons for these unintended pregnancies were school closures, limited access to birth control and health services, and more sexual activity.

The current evidence is limited. More research is needed to understand the full impact of school closures and lockdowns on young people and to find ways to safeguard them from further crises.

**Supplementary Information:**

The online version contains supplementary material available at 10.1186/s12978-025-02045-7.

## Introduction

### Background

The global response to the COVID-19 pandemic included lockdown measures such as school closures [1]. These measures significantly impacted education systems, with school closures affecting almost 90% of students worldwide [2]. Across the world, prolonged COVID-19 lockdowns had multiple indirect negative effects on numerous health outcomes, including mental and physical well-being [1]; additionally, causing further inequality through economic and social consequences among disadvantaged groups such as young people and women [3, 4]. In low-and middle-income countries (LMICs), the existing challenges to health and well-being have been aggravated manifesting in e.g. disrupted education [1], compromised sexual and reproductive health and rights (SRHR) [5], and a rise in unintended pregnancies among adolescent girls and young women (AGYW) [6].

Interrupted schooling may have a negative effect on young people’s human capital, i.e., a person’s accrued health and knowledge [1]. While several governments worldwide implemented distance learning as a way to ensure continued schooling, a lack of technical and infrastructural resources and direct teacher-student interaction made it challenging to ensure continued education and prevent dropouts [7]. This may have exacerbated existing inequalities and learning gaps between rich and poor, in particular in LMICs [7]. The magnitude of the education crisis following the pandemic has not yet been fully measured, with millions of students at risk of postponing their return to school or dropping out due to various reasons [1, 8]. World Bank data shows that at least one billion children in LMICs missed at least one year of in-person schooling, resulting in significant learning losses [1] and lack of safe spaces particularly for girls [9]. School closures may therefore be particularly harmful to girls who may be exposed to higher risks of, early marriage, unintended pregnancy, sexually transmitted infections and gender-based violence [10]. The negative effects can be life-long as young pregnant girls often discontinue their education and drop out of school which affects their future economic prospects and may reinforce overall poverty levels [11, 12].

The global adolescent birth rate (ABR) (15–19 years) has been on a steady decline from 64.5 births per 1,000 women in 2000 to 41.3 in 2023 [13]. However, there are large regional and national differences, the ABR in WHO Europe region is about 13 births per 1,000 women compared to 97 in the WHO Africa region. Specific groups, including those with lower education or economic status, are experiencing a slower decline in the ABR, resulting in greater economic and educational inequities [13]. At 100 births per 1,000 women in 2021, sub-Saharan Africa has the highest adolescent birth rate globally [14]. Approximately half of the pregnancies among 15–19-year-olds in LMICs are unintended, potentially resulting in risky and unsafe abortions [15]. The causes of adolescent pregnancies in LMICs are diverse, stemming from societal pressure for girls to marry and have children, as well as limited autonomy of married girls in childbearing [13]. Other causes are limited educational and employment prospects for girls [13]. Unintended pregnancies not only lead to mortality and morbidity for the young mother and children [13], but also perpetuate cycles of poverty and reinforce social and economic inequalities that often persist into the next generation [16].

In 2021, the United Nations Population Fund (UNFPA) projected the disruption of contraceptive services during the pandemic for 12 million women could result in up to 1.4 million unintended pregnancies across 115 LMICs during the first year alone [17]. Estimates and predictions of the potential impact of COVID-19 lockdowns on unintended pregnancies have been made, indicating potentially large increases [18]. However, a mapping and review of the evidence of the impact of COVID-19 lockdowns on unintended pregnancies among girls and young women in LMICs has not been conducted.

This scoping review aims to identify and describe the available evidence on the impact of COVID-19 lockdown measures on unintended pregnancies among adolescent girls and young women, aged 10–24 years, across LMICs. By synthesizing findings from multiple countries, we aim to identify overarching themes and trends rather than granular, country-specific analyses. Recognizing that reported increases in unintended pregnancies to some extent may reflect population perceptions rather than actual trends, this review seeks to explore existing literature to the identification of research gaps and inform policy directions.

### Definition of key terms

We adopt the definition of “unintended pregnancy” suggested by UNFPA [4]. As such, an unintended pregnancy is “a pregnancy that occurs to a woman who was not planning to have any (more) children, or that was mistimed, in that it occurred earlier than desired”. A synonym for unintended pregnancy is unplanned pregnancy [5]. An unwanted pregnancy, on the other hand, is “a pregnancy that a woman does not want to have”; it occurs when a woman does not want to have any children at all, or any more children [5].

## Methods

In order to provide a broad overview of the effects of COVID-19 lockdowns on unintended pregnancies among AGYW across LMICs, this scoping review adhered to the Joanna Briggs Institute Manual for Evidence Synthesis, the five-stage framework by Arksey and O´Malley following recommendations given by Khalil et al. [19, 20]. The objectives, inclusion criteria and methods for this review were outlined in an internally developed protocol to fill the existing knowledge gaps. The aims of this scoping review include the identification and description of key concepts connected to the research topic [21]. By identifying and describing existing evidence on COVID-19 lockdowns and their potential impact on unintended pregnancies, this review offers a nuanced understanding of how lockdown measures intersected with existing barriers to SRH access. Furthermore, it helps to determine the extent of the available literature, identify gaps, summarize existing evidence, and potential areas for future research [21].

### Search strategy and selection criteria

To ensure identification of all evidence (published scientific articles and grey literature) on the impact of the COVID-19 lockdown measures on SRHR of AGYW, focusing on unintended pregnancies, we followed a three step process outlined by Peters et al. (2015) and the Joanna Briggs Institute Manual for Evidence Synthesis [19]. Firstly, key papers and reports were provided by the second and last author. This “pearl harvesting” based on Waddington et al. [22], supported the determination of initial keywords for the search. Secondly, with the support of an expert librarian and the previously collected keywords, based on the PICO criteria (patient/population, intervention, comparison and outcomes), we conducted a systematic and comprehensive search in July of 2023 on CABI (a global health digital library—https://www.cabidigitallibrary.org/product/he). An updated search, using the same search string, was performed on 27 th May 2024 on LUBsearch (a search engine at Lund University covering some 100 subject databases—https://www.lub.lu.se/en/find/lubsearch). This approach adhered to the recommendations by Peters et al. which include conducting an initial limited search on a minimum of two pertinent online databases aligning with the subject matter [23]. The search string comprised a combination of the keywords “COVID-19”, “lockdown”, “unintended pregnancies” and “SRHR”, along with relevant synonyms. The full search strategy can be found in additional file 1. Furthermore, websites of selected organizations (see additional file 1 for details) were searched comprehensively, combining the keywords “unintended pregnancy” and “COVID-19” to identify grey literature. The publication filter for studies between 2019 and 2024 was applied. Thirdly, the reference lists of studies identified during the database search were screened for additional relevant studies.

Publications were included if they were published between 2019 and 2024, written in English, the study setting was low- or middle-income countries according to the Organization for Economic Co-operation and Development (OECD) classification [24], the outcome was concentrated on SRHR, with a specific focus on (unintended, unwanted, teenage) pregnancies. Furthermore, publications were included if the intervention included COVID-19 pandemic lockdown measures, such as quarantine, confinement, restrictions and school closures. Only original research publications with a quantitative or qualitative research design and grey literature that employed quantitative or qualitative methods, were deemed eligible for inclusion. Commentaries, opinions and reviews were excluded. However, reference lists of review articles were screened and original studies cited in review articles were included. We used Covidence, a web-based software for conducting reviews, to organize, manage, and screen the articles, including removing duplicates. The first author screened all publications based on the inclusion criteria. Uncertainties regarding the inclusion of studies were resolved through discussions within the research team.

### Data charting, collation, summarizing and reporting results

Data were extracted using a standardized charting tool in Covidence. The Data Extraction Template (see additional file 2) was inspired by a chapter about Data Extraction/Data Charting in the Joanna Briggs Institute Manual [19] and Extracting and Charting the Data from Khalil et al. (2016). It was utilized to assemble key characteristics relevant to the review objectives, including publication year, design, setting, target population, sample size, study duration and main results. Data was charted by one reviewer. The process of data charting provided guidance for the data summarizing development. In accordance with the review objectives, we conducted a descriptive analysis of the scope, characteristics and distribution of the included publications. While the review primarily focused on mapping the evidence rather than performing an in-depth subgroup analysis, the findings highlight trends across different LMIC contexts. The descriptive findings are presented in figures and tables.

## Results

### Search and selection of publications

**Fig. 1:**
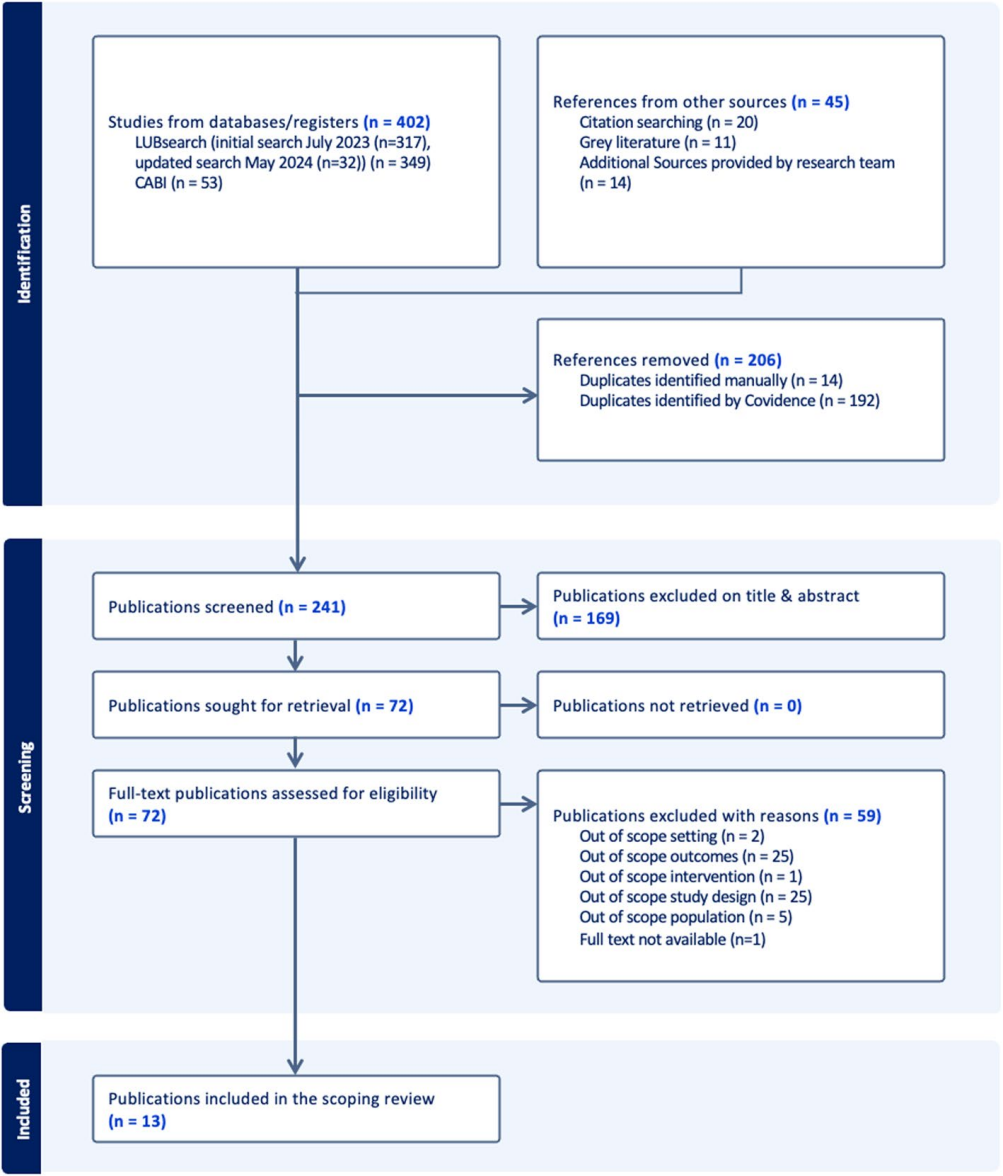
Flow Diagram Selection of Publications. This figure shows a flow diagram detailing the publication selection process

LUBsearch initially yielded 464 and CABI 53 results. After removing duplicates, a total of 370 results were uploaded to Covidence (317 from LUBsearch and 53 from CABI). An additional 14 citations, provided by the last author, were included in the screening. These 14 publications were identified in the initial phases of the study. Furthermore, 20 publications were identified following a manual search of the reference lists of relevant reviews and publications provided by the supervisors and included studies. Eleven grey literature publications were included in the screening. In the updated LUBsearch in May 2024, 32 studies were identified, bringing the total number of studies to 349 from LUBsearch. After deduplication, 241 publications were screened for relevance and 72 studies met the eligibility criteria based on title and abstract and were included for full text review. After the full text assessment, 13 publications were included in the scoping review. Figure 1 shows the PRISMA flowchart illustrating the search process.

### Characteristics of included publications

**Fig. 2:**
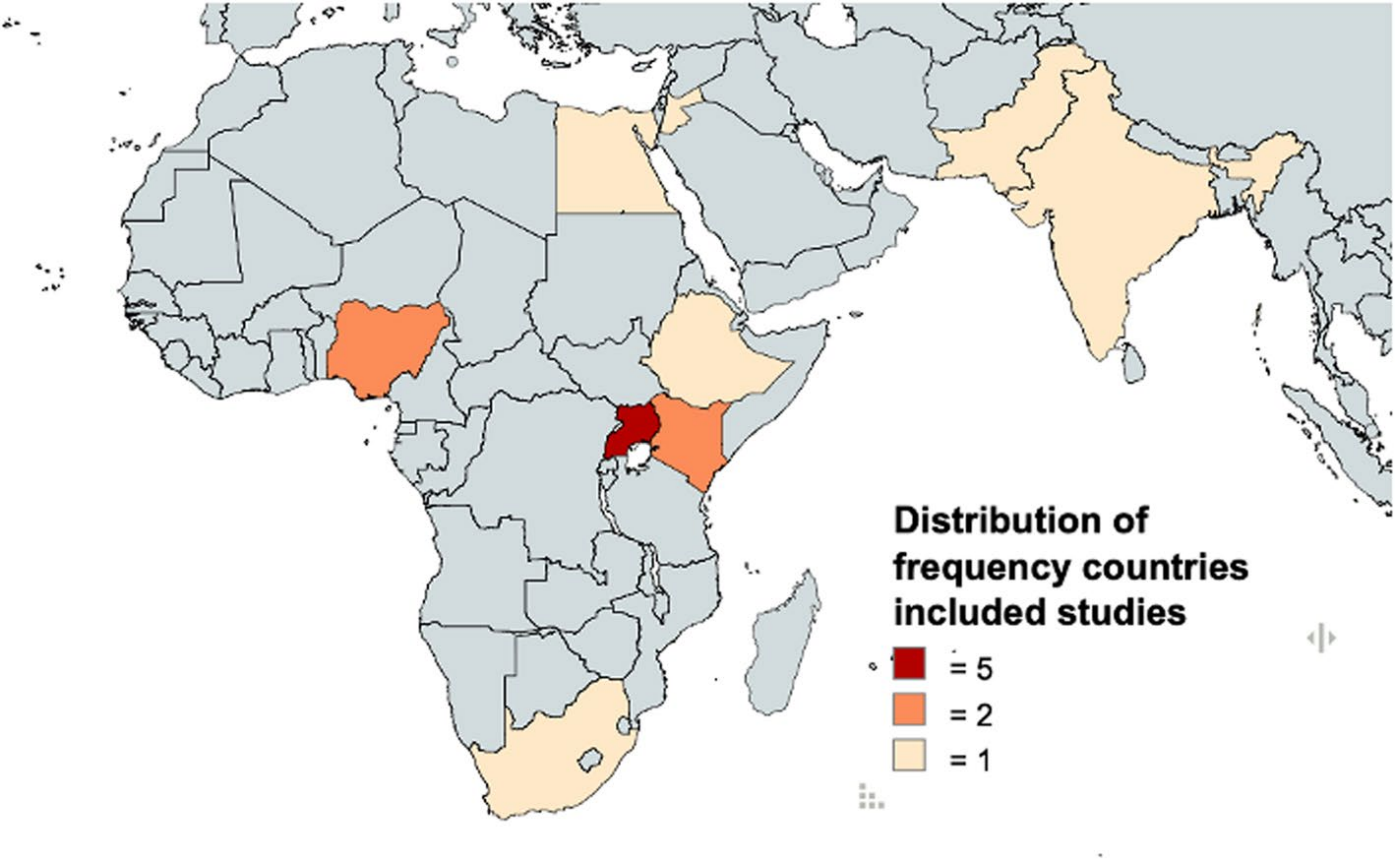
Visualization of country distribution of included studies. This figure shows a visualization of the distribution of the frequency of countries among the included studies

The general publication characteristics are presented in Table 1. Included articles were published between 2021 and 2024. The studies were conducted in the following countries: Uganda (*n* = 5), Nigeria (*n* = 2), Kenya (*n* = 2), South Africa (*n* = 1), Lebanon (*n* = 1), Ethiopia (*n* = 1), India (*n* = 1), Egypt (*n* = 1) and Pakistan (*n* = 1) (Table 1 and Fig. 2). Most publications used quantitative methods (*n* = 7), qualitative (*n* = 4) and the remaining mixed methods (*n* = 2). The sample size varied from 46 to 6394 participants. The criteria for defining unintended pregnancy differed between the studies, with the majority not following established global definitions. Some studies measured binary (intended or unintended) outcome while others measured the intention under three categories (mistimed, wanted and unwanted). Four studies applied the classification of “unintended/unplanned pregnancy”, three used “teenage pregnancies”; two used the term “adolescent pregnancy”, two used the term “unwanted pregnancy” and the two remaining studies applied each two classifications (unintended and adolescent pregnancy and unintended and teenage pregnancy) for the occurrence of pregnancy during COVID-19 lockdowns.

**Table 1:** General characteristics of included publications and distribution of frequencies of countries

General characteristics of included studies (*n* = 13)		
	**Number**	**Share (%)**
**Publication Year**		
2021	1	8%
2022	11	84%
2024	1	8%
**Publication Type**		
Journal Article	10	77%
Grey Literature	3	23%
**Scope**		
Single country	12	92%
Multi-country	1	8%
**Design**		
Quantitative	7	54%
Qualitative	4	31%
Mixed Methods	2	15%
**Classification/Measurement of Pregnancy**		
Teenage Pregnancy	3	23%
Unintended/unplanned Pregnancy	4	31%
Adolescent pregnancy	2	15%
Unwanted Pregnancy	2	15%
Multiple classifications	2	15%
**Distribution of frequency of countries – included studies**		
**Country**	**Frequency**	
Uganda	5	
Nigeria	2	
Kenya	2	
South Africa	1	
Lebanon	1	
Ethiopia	1	
India	1	
Egypt	1	
Pakistan	1

### Summary of evidence

This review analyzed 13 publications from 9 different LMICs, covering various scopes, designs, and publication types. The evidence indicates a (perceived) increase in unintended and teenage pregnancies among AGYW in LMICs during the COVID-19 pandemic, with pregnant girls being more likely to drop out of school.

### Effects of COVID-19 lockdown on pregnancy

The focus of this scoping review is to provide an overview of the effects of COVID-19 lockdowns on unintended pregnancies among AGYW across LMICs. The evidence shows either an increase in numbers of (unintended, teenage or unwanted) pregnancies among AGYW during the lockdown or a qualitatively assessed perceived rise in unintended pregnancies. Additional file 3 provides a summary of the first author, country, sample size, classification of pregnancy and evidence of reported or perceived levels of pregnancies during COVID-19. The table in additional file 4 provides more detail including study design, study population and share of female participants.

### Evidence of changes in the prevalence of unintended pregnancies during COVID-19

#### Age group 10–24 years

The evidence for the age group 10–24 years reveals a marked increase in pregnancies among AGYW during COVID-19, with rises reported across various publications and country settings. These findings highlight the heightened vulnerability of adolescents to unintended pregnancies during COVID-19. In the following sections these findings are described in more detail.

A study in Hoima District, Uganda on the frequency and variables associated with adolescent (13 to 19 years) pregnancy among female students during the pandemic, revealed that 30% of teenage girls became pregnant during this period, which was an increase [25], compared to the pre-COVID-19 period when 25% of all teenage girls became pregnant [25].

A study by the Forum for African Women Educationalists (FAWE) Uganda Chapter also yielded valuable insights into the incidence of pregnancy among AGYW during the 2-year long school lockdown [9]. Using a cross-sectional survey, a structured questionnaire and focus group discussions (FGDs) (12 FGDs with parents/caregivers and at least 12 FGDs with groups of 5–10 girls, boys and young women and men), FAWE found a 22.5% increase (from 80 655 to 98 810 cases) in pregnancies among girls seeking initial antenatal care between March 2020 and June 2020 [9]. The highest increase in pregnancies occurred among girls aged 10–14 years which rose by 366.5% (from 290 in March to 1 353 cases in September 2020). In comparison, pregnancies among those aged 15–19, increased by 25.2% and among young women aged 20–24 by 21.1%. Moreover, 50% young individuals, reported a perceived rise in the number of girls becoming pregnant during the COVID-19 period, as compared to pre-pandemic times [9].

Zulaika et al. studied the incidence of pregnancy among adolescent girls attending secondary school in Kenya. The results show that not attending school for 6 months because of COVID-19 lockdowns, led to twice the risk of pregnancy among adolescent secondary schoolgirls, compared to their counterparts who graduated prior to the outbreak. Specifically, the occurrence of pregnancies between the start of Form 3 and completion of examinations was recorded at 10.9% in COVID-19 girls’ cohort versus 5.2% in the pre-COVID-19 cohort [6].

The wellbeing of children in Uganda amidst the COVID-19 pandemic has been researched by the AfriChild Center in Uganda who analysed data obtained from the Uganda Child Help Line (https://sauti.mglsd.go.ug/sauti/). The analysis revealed a rise of teenage pregnancies from April to September 2020 compared to 2019. In August 2019, 7 cases on teenage pregnancy were reported compared to 24 cases in August 2020. Furthermore, FGDs with 31 caregivers and parents indicate a perceived escalation in teenage pregnancies among girls who are out of school due to lockdown measures [26].

A study by Mambo et al. [27] provides insight into the obstacles that young individuals (most aged 18–24) encountered when trying to access and utilize SRHR services during the pandemic-induced lockdown in Uganda of whom 43.6% were females. The results indicate that unwanted pregnancies were the second most prevalent SRHR issue (32.4%) faced by Ugandan youth during the lockdown [27].

A qualitative study by Adelekan et al. explored women´s (aged 15—20 and above) experiences of access to SRH services during the COVID-19 lockdown in Nigeria through ten FGDs, and found a perceived rise in unplanned pregnancies, particularly concentrated among unmarried adolescents [28].

#### Age group 18 years and above

The evidence for the age group 18 years and above collectively indicate a rise in unintended and teenage pregnancies during the COVID-19 pandemic across various regions. These findings show the adverse impact of COVID-19 related disruptions of SRH services on unintended pregnancies.

A study by Khan et al. provides a qualitative analysis of the perceived unintended consequences of COVID-19 health measures on youth SRH in two rural districts in Uganda. One of the outcomes also assessed unintended and teenage pregnancies. All participant groups expressed concerns regarding the public health strategies implemented in those districts in Uganda, which were perceived to have contributed to surges of pregnancies and other related outcomes [29].

Elsaid and colleagues retrospectively investigated the effects of the lockdown on sexual function and reproductive health among 409 married women in Egypt aged 18 and above. Almost half of the study population (45.5%) became pregnant during the pandemic, among which only 30% were described as intended or wanted [30].

A prospective cross-sectional study conducted by Mustafa et al. evaluated the contraceptive usage and occurrence of unplanned pregnancies among 350 married women of reproductive age in Pakistan during the pandemic. One key result showed that 26.6% of women reported being pregnant, out of which 80.6% described their pregnancies as unplanned [31].

In a cross-sectional study, Haddad et al. investigated factors associated with pregnancy status and pregnancy intention (wanted/unwanted) among Lebanese women aged 18 to 51 during the pandemic and found that 11.1% reportedly became pregnant during the pandemic, with 22% of those pregnancies being described as unwanted [32].

A study by Tenaw et al. measured the incidence of unintended pregnancy and related factors among women who received antenatal care in public hospitals in Southwest Ethiopia during the COVID-19 pandemic. Unintended pregnancy occurred among 19.5% of the participants. Out of these pregnancies, 50.6% were described to be mistimed and 49.4% as unwanted. The majority (54.1%) of the 15 to 19-year-old participants, accounting for 5.9% of the total study population, reported unintended pregnancies. Similarly, among the 20–34-year old, comprising 72.3% of the total, 14.6% experienced unintended pregnancy [33].

A qualitative study by Chimbindi et al. explored the impact of COVID-19 on the SRH needs of school-going youth in rural KwaZulu-Natal, South Africa. The study findings show a perceived rise in early pregnancies among students during the COVID-19 pandemic [34].

In a Women Deliver report [35] based on interviews and FGDs with key informants and youth advocates from India, Kenya and Nigeria, respondents reported that consequences of the pandemic such as disrupted access to SRH services have resulted in an increase of unintended pregnancy [35].

### Factors associated with unplanned pregnancies during COVID-19

Some of the included studies analysed additional factors linked to unplanned pregnancies amid the COVID-19 pandemic, including the school closures.

Chimbindi et al. (2022) showed that the implementation of COVID-19 lockdown measures, including school closures and lack of SRH education and contraception opportunities, resulted in pregnancy increases among school girls, often leading to school dropout in South Africa [34]. Similarly, Khan et al. (2022) and Adelekan et al. (2024) highlighted how the rising numbers of unemployed and out-of-school youth during the pandemic has contributed to increased sexual activity. Furthermore, the study by Zulaika et al. (2022) highlights that the pandemic in Kenya prompted nationwide curfews, lockdowns and restrictions of movement, resulting in school closures for six months. The findings suggest that girls exposed to COVID-19 lockdown measures were three times more likely to drop out of school. Moreover, they had a 3.4 times higher risk of school transfer prior to examinations compared to students in the pre-COVID-19 era. Furthermore, the study suggests that the containment measures in Kenya have exacerbated the negative SRH and education outcomes for adolescent girls, thereby increasing the risk of unintended pregnancies [6].

Almost half (43.9%) of the participants in the FAWE study [9] expressed that sexual violence against girls had escalated during school closures due to children aimlessly wandering, thereby exposing themselves to heightened levels of alcoholism, substance abuse, bad company, and sexual violence. Approximately 22.2% of female respondents reported that certain girls have been forced to engage in transactional sex due to impoverishment within their household. Out of the girls expressing their intention to not return to school, 9.8% mentioned “pregnancy” as a reason. Additionally, when school records were scrutinised, the FAWE researchers discovered that more females had abandoned their studies in candidate classes compared to their male counterparts upon the reopening of schools [9].

The AfriChild (2021) article, based on FGDs with 31 caregivers and parents, showed that the school closures also impacted the involvement of children in paid labour. This can have repercussions when boys engage in paid work and utilize their earnings to entice girls into sexual relationships, leading to unwanted pregnancies. The findings of the FGDs reveal that parents expressed concerns regarding the reintegration of schoolgirls who became pregnant during the COVID-19 lockdown, into the education system. Their concern primarily revolved around stigma associated with teenage pregnancy potentially further exacerbating the drop out of girls from school [26].

Other factors associated with unplanned pregnancies among AGYW were widely distributed. The FAWE study (2023) and Adelekan et al. (2024) examine the difficulties encountered by AGYW in accessing SRH services during the confinement period, which may have contributed to unintended pregnancies. These obstacles comprise limited availability of and access to contraceptives, restricted movement due to lockdown measures, and apprehension of contracting COVID-19 at healthcare facilities [9]. Furthermore, it was also found e.g. in Nigeria that unmarried adolescents wanting to use contraceptives, are often discriminated against and therefore experience unintended pregnancies and early marriage as a result [28].

The study by Musinguzi et al. studied the frequency and variables linked to adolescent (13 to 19 years) pregnancy among female students during the COVID-19 pandemic period. Their study showed that factors associated with increased rates of teenage pregnancies during the pandemic encompassed being unmarried, attending boarding schools, abstaining from contraceptive use and engaging in sex trade [25].

## Discussion

### Societal consequences of school dropout

This review identified and analysed 13 publications from 9 different LMICs, including varying country scope, design and publication types. The findings were then divided in two different population groups, including 10–24-year-olds and 18 years and above. The evidence included in this scoping review show an increase in unintended and/or teenage pregnancies during the COVID-19 pandemic among AGYW in LMICs. Furthermore, girls becoming pregnant during COVID-19 were more prone to school dropout [6]. However, it needs to be acknowledged that qualitative findings in this review are primarily based on participants'perceptions.

This increase in pregnancies among students during the COVID-19 lockdown may seem counterintuitive given the social distancing restrictions. Nevertheless, among the factors associated with unintended pregnancies during COVID-19 were school closures, limited access to SRH services including contraceptives and increased sexual encounters/relationships [29]. School closures expose AGYW to teenage and unintended pregnancies but also to other risk factors for unintended pregnancies such as sexual violence, child marriages, exploitation and abuse [36] and can lead to an increase in idle youth and limited opportunities to learn about SRH in schools [34]. Nevertheless, it can be inferred that lockdown measures, including school closures, had a detrimental effect on AGYW in LMICs, and pregnancy can be interpreted as both a cause and a consequence of school dropout [5, 35].

During the Ebola outbreak in West Africa in 2013 for example [37], it was shown that the occurrence of a pregnancy may act as a catalyst for girls, particularly those who are economically disadvantaged or already lagging behind their education, to drop out of school [38]. The Ebola outbreak in sub-Saharan Africa also prompted school closures and resulted in a surge in teenage pregnancies and a decrease of 17% in school enrolment among young girls following the reopening of schools [39]. These findings are corroborated by another article, demonstrating that pregnancies were the leading cause of dropout among girls in secondary school, accounting for 40.2% of cases, pre COVID-19 [40]. Despite the statistics regarding the prevalence of high birth rates among adolescents in Sub-Saharan Africa presented by the World Health Organization (WHO) [13], the general level of protection afforded to pregnant girls in the region concerning their right to education varies significantly [41].

The challenges faced by adolescent girls extend beyond legal barriers, including social issues such as stigma and discrimination from peers, educators and community members [38, 42]. A notable example of this is in Sierra Leone, where the Minister of Education prohibited visibly pregnant girls from re-enrolling in schools following the reopening of educational institutions post-Ebola outbreak [43].

It is important to mention that the differences in contexts and settings of publications included in this review highlight the importance to interpret the presented findings with greater caution. Subgroup analyses such as stratification by location, religion, or income, were not conducted due to the different character of the studies included. For example, implications of unintended pregnancies might differ significantly among AGYW who are married or unmarried [44]. Furthermore, culture and religion can shape how communities perceive or respond to pregnancies in e.g. AGYW [45].

Another essential note is to be taken on the fact that unintended pregnancies are not uniformly perceived as unwanted. Often, AGYW in LMICs experience unintended pregnancies resulting from (among others) social norms, a lack of SRH services and systemic inequalities, whereas in other settings AGYW might have had greater autonomy about pregnancies, even during the COVID-19 pandemic.

### Economic consequences of school dropout

The review revealed some evidence of an increase in unintended pregnancies and school dropouts among AGYW. This is particularly worrying as the consequences of AGYW´s school dropout due to unintended pregnancies include limited educational attainment, loss of human capital and a reduction in lifetime earnings [46]. Keeping girls in school and investing in their education contributes greatly to their own and their children´s health and well-being, as well as protecting them from discrimination, exploitation and abuse [47]. Furthermore, educated girls earn higher incomes and their odds of marrying young are lower [48]. Thus, loss of schooling does not only have implications for the individual girl or young women affected but may also influence the economic and societal growth.

Individuals under the age of 25 who were affected by the pandemic are projected to comprise a significant 90% of the working population in their prime years by 2050 [40]. The option to delay pregnancies until a more advanced age is not only beneficial from the reproductive health perspective in general but also will potentially contribute to the enhanced economic and societal empowerment of young women who can either pursue further education or secure financially more rewarding employment opportunities [49].

Using a conceptual framework, Chaaban and Cunningham measured the opportunity cost associated with the exclusion of girls from productive employment, prior to the pandemic. The methodology used in their article was originally developed by the World Bank. It was employed to calculate the economic costs encountered because of the exclusion of girls and young women, with a focus on early school dropout, unemployment and pregnancy [49]. The analysis included 14 countries (Bangladesh, Brazil, Burundi, China, Ethiopia, India, Kenya, Malawi, Nigeria, Paraguay, Senegal, South Africa, Tanzania, and Uganda) and findings revealed that investing in girls has the potential to result in significant economic benefits for countries. Investments in social inclusion of adolescent girls to provide them with the means they need to reach their full potential, can result in substantial economic growth [49]. The approach included a simple non-parametric methodology to achieve an approximate quantification of the costs incurred by societies due to the exclusion of adolescent girls. The results focus on the opportunity cost, measuring the losses in terms of potential gains in productivity and income that young girls could have achieved if they were employed, delayed pregnancy or achieved higher levels of education. The findings suggest that even small investments in girls can have a significant impact on both GDP growth and on overall well-being [49]. Hence, the COVID-19 pandemic has likely aggravated these negative effects of girls dropping out of school.

A publication by Schady et al. (2023) evaluated the consequences of the COVID-19 pandemic on education and human capital of youths in LMICs. The publication examined the economic consequences of the pandemic on future earnings potential of the affected cohort of students [1]. The results indicate that the pandemic has had negative effects on education and human capital, particularly on the group of disadvantaged students. Even though some increases in school enrolment after the pandemic were observed, these were dominated by the corresponding deteriorations in employment, suggesting that more youths were neither studying nor working compared to what would have been expected without the pandemic [1]. The closure of schools due to the pandemic may result in reduced years of schooling for some youths. Moreover, the surge in numbers of school dropouts in LMICs during the pandemic is also attributable to school closures. The decrease in school enrolment was comparable for both male and female students but considerably bigger for children from low-education households [1]. The authors summarize that the eventual reopening of schools could only partly alleviate the consequences of the school closures. Those consequences include the significant decline of social and emotional skills and executive functions of the children. Lastly, the authors calculate the economic ramifications of the pandemic regarding the future income losses for the affected student cohort. The pandemic induced educational setbacks may lead to a 1.5% decrease in lifetime earnings, amounting to an estimated loss of $872 billion for the global economy [1].

### Limitations

A comprehensive scoping review methodology was employed to identify evidence on this important topic, complemented by grey literature search in selected databases. Limitations include exclusion of languages other than English. In addition, there was a relatively small number of eligible studies identified for inclusion and the studies mostly used qualitative design including perception-based responses which limit reliability and generalizability of the evidence. Nonetheless, given the usual challenges of the LMIC contexts, the identified evidence offers valuable knowledge in the area. This foundational knowledge can guide researchers in an important direction employing more robust study designs. Socioeconomic status data was not extracted for this review. However, this data can offer valuable insights, and these links should be explored in future research to better inform interventions. Furthermore, this scoping review does not include a quality assessment of the included studies and presented only descriptive analysis. Additionally, the data is time-limited to articles published within a specific window and therefore, the long-term consequences on increased unintended pregnancies remain unclear. Classifications and definitions for pregnancies varied across publications, with many not referencing established definitions, limiting comparability.

## Conclusion

This review has shown that there is some evidence to suggest that the COVID-19 lockdowns in some LMICs have contributed to an increase in unintended and unwanted pregnancies among AGYW. However, the review also revealed that current evidence is limited and may lack reliability in several aspects. The closure of schools contributed to a major disruption of access to SRHR education, and services thus highlighting the vital role of schools in health promotion and social protection among AGYW.

Overall, robust evidence from the COVID-19 pandemic is lacking, specifically for outcomes such as teenage pregnancy including often not using a robust and established definition. More vigorous research with sub-group analysis such as urban/rural setting, SES and religion are needed to detect and understand the magnitude of the pandemic among this population. Moreover, due to the variety of contexts and cultural diversity it is important to interpret the findings considering the local contexts. Furthermore, it is essential to conduct long-term research and explore the possible occurrence of unintended and teenage pregnancies [1].

In conclusion, the studies emphasize the complex magnitude of the COVID-19 pandemic on unintended and teenage pregnancies. The ramifications not only affect individuals, but also the global economy and labor force with significant direct and indirect costs. Young girls leaving school following an unintended pregnancy led to restricted educational attainment and a depletion of human capital, holding back economic and social empowerment. This was exacerbated during the pandemic.

Dedicating more resources to adolescent girls' development, alongside implementing targeted social interventions—such as strategies for stigmatized or bullied youth—can have significant economic advantages for countries by increasing income, productivity, and overall well-being, to avert the current consequences, especially during future pandemics.

## Supplementary Information

Additional file 1. Elaboration of methods, search string, databases and grey literature search*.*

Additional file 2. Data Extraction Template

Additional file 3. Table - Summary of included publications*.*

Additional file 4. Table - Detailed overview of included publications.

## Data Availability

No datasets were generated or analysed during the current study.

## Abbreviations

LMICs: Low-and middle-income countries
SRH: Sexual and reproductive health
SRHR: Sexual and reproductive health and rights
AGYW: Adolescent girls and young women
ABR: Adolescent birth rate
UNFPA: United Nations Population Fund
LUB: Lund University Bibliography
OECD: Organization for Economic Co-operation and Development
ODA: Official Development Assistance
FAWE: Forum for African Women Educationalists
FGDs: Focus group discussions
WHO: World Health Organization
