# Supplementary material for: Effects of COVID-19 lockdowns on unintended pregnancies among adolescent girls and young women in low- and middle-income countries: a scoping review
Source: Reprod Health. 2025 May 22;22:89. doi: 10.1186/s12978-025-02045-7 (PMC12096587; doi:10.1186/s12978-025-02045-7)
Supplement: Supplementary file 1 — Additional file 1. Elaboration of methods, search string, databases and grey literature search. [file 12978_2025_2045_MOESM1_ESM.docx]

Search String used in LUBsearch – 7 July 2023

(Covid-19 OR coronavirus OR SARS-cov2) AND (lockdown* OR quarantine OR confinement OR restriction*) AND ((Unintended OR unplanned OR unwanted)) AND pregnan* OR (srhr OR "sexual and reproductive health and rights" OR "sexual and reproductive health")

Databases and results from LUBsearch

| **Database** | **Hits** |
| --- | --- |
| MEDLINE | 120 |
| Directory of Open Access Journals | 71 |
| Academic Search Complete | 59 |
| Complementary Index | 56 |
| CINAHL Complete | 37 |
| Springer Nature Journals | 26 |
| Scopus® | 19 |
| APA PsycInfo | 12 |
| Supplemental Index | 12 |
| JSTOR Journals | 7 |
| Social Sciences Citation Index | 6 |
| LGBTQ+ Source | 5 |
| Science Citation Index Expanded | 5 |
| ScienceDirect | 5 |
| Library catalogue (LUBcat) | 4 |
| Springer Nature eBooks | 4 |
| Criminal Justice Abstracts with Full Text | 3 |
| SocINDEX with Full Text | 2 |
| Business Source Complete | 2 |
| SwePub | 1 |
| APA PsycArticles | 1 |
| Urban Studies Abstracts | 1 |
| Philosopher's Index | 1 |
| GreenFILE | 1 |
| Arts & Humanities Citation Index | 1 |
| British Library EThOS | 1 |
| ePublications | 1 |
| Books at JSTOR | 1 |
|  | 464 total |

Sources LUBsearch - 7 July 2023

List of all databases included in LUBsearch can be found here: <https://emedia.lub.lu.se/db/all>

Grey literature database search – 20 July 2023

keywords used: unintended pregnancy and COVID.

publication filter (if possible): 2019-2023

- World Bank
- UNFPA
  - <https://www.unfpa.org/search/content?search_api_fulltext=unintended%20pregnancy%20and%20covid&page=1>
- WHO Regional Office Africa
  - <https://www.afro.who.int/search/google-cse?keys=unintended+pregnancy+and+covid>
- UNICEF
  - <https://www.unicef.org/media/94016/file/The-importance-of-investing-in-the-wellbeing-of-children-to-avert-the-learning-crisis.pdf>
- UN Women
- European Commission
