# Supplementary material for: Effects of COVID-19 lockdowns on unintended pregnancies among adolescent girls and young women in low- and middle-income countries: a scoping review
Source: Reprod Health. 2025 May 22;22:89. doi: 10.1186/s12978-025-02045-7 (PMC12096587; doi:10.1186/s12978-025-02045-7)
Supplement: Supplementary file 2 — Additional file 2. Data Extraction Template [file 12978_2025_2045_MOESM2_ESM.docx]

| **Study information** | **Options** |
| --- | --- |
| Title |  |
| Author(s) |  |
| Year of publication | Single choice   - 2019 - 2020 - 2021 - 2022 - 2023 - 2024 - Other |
| Language | - English |
| Journal/Source |  |
| Study Characteristics | Multiple choice   - Quantitative - Qualitative - Cross-sectional - Cohort - Case-control - Survey - Interviews - Grey literature - Other |
| Sample size |  |
| Country/Setting | Multiple choice   - LMIC - Other |
| Study duration |  |
| Participant characteristics | Age range (multiple choice)   - Adolescent girls (10-19) - Young women (20-24) - Both (10-24) - Other |
| Other relevant demographics |  |
| Outcomes | Multiple choice  SRHR   - Unintended pregnancies - teenage/early pregnancies - other |
| Intervention | Multiple choice   - COVID-19 lockdown measures - Quarantine/confinement - School closures - Other |
| Key findings/results |  |
| Authors conclusions and implications |  |
| Additional notes/comments |  |
