# Supplementary material for: Effects of COVID-19 lockdowns on unintended pregnancies among adolescent girls and young women in low- and middle-income countries: a scoping review
Source: Reprod Health. 2025 May 22;22:89. doi: 10.1186/s12978-025-02045-7 (PMC12096587; doi:10.1186/s12978-025-02045-7)
Supplement: Supplementary file 3 — Additional file 3. Table - Summary of included publications. [file 12978_2025_2045_MOESM3_ESM.docx]

|  | **First author** | **Country** | **Sample Size** | **Classification of pregnancy** | **Outcomes of Interest** |
| --- | --- | --- | --- | --- | --- |
| 1 | Adelekan  et al | Nigeria | 94 | Unplanned pregnancy | Increase in unplanned pregnancies resulting from COVID-19 lockdown |
| 2 | AfriChild | Uganda | 1 800 | Teenage pregnancy | Increase in teenage pregnancies; 8 cases in April 2019 vs 23 in September 2020 in sample |
| 3 | Chimbindi et al | South Africa | 69 | Teenage pregnancy | Perceived increase in teenage & early pregnancies among learners; school drop out |
| 4 | Elsaid | Egypt | 409 | Unwanted pregnancy | 45.5% participants reported pregnancies; 70% of pregnancies reported to have been unintended |
| 5 | FAWE | Uganda | 6 394 | Adolescent pregnancy | 22.5% increase in pregnancy among girls between March and June 2020; highest increase among girls aged 10-14 (366.5%) compared to girls aged 15-19 (25.5%) and young women aged 20-24 (21.1%) |
| 6 | Haddad et al | Lebanon | 369 | Unintended pregnancy assessed using binary question | 22% reported unwanted pregnancies |
| 7 | Khan et al | Uganda | 94 | Unintended & teenage pregnancy | Perceived increase in youth pregnancy due to school closures |
| 8 | Mambo et al | Uganda | 733 | Unwanted pregnancies | Unwanted pregnancy one of the most common SRH problems reported during COVID-19 lockdown (32.4%); |
| 9 | Musinguzi et al | Uganda | 314 | Teenage pregnancy | 3 out of 10 teenage girls got pregnant during COVID-19; slight increase in teenage pregnancies |
| 10 | Mustafa | Pakistan | 350 | Unplanned pregnancy | 26.6% of women reported pregnancies; 80.6% out of those reported these to have been unplanned |
| 11 | Tenaw et al | Ethiopia | 421 | Unintended pregnancy | 19.5% prevalence of unintended pregnancy; 50.6% mistimed and 49.4% unwanted; below 20-year-olds: 54.1% experienced unintended pregnancy |
| 12 | Women Deliver | India, Kenya & Nigeria | 46 | Unintended and adolescent pregnancy | Perceived increase in unintended pregnancies as result of COVID-19 lockdown and reduced access to family planning |
| 13 | Zulaika et al | Kenya | 910 | Adolescent pregnancy | School disruption leading to two times the risk of adolescent pregnancy in the COVID-19 cohort |
