# Supplementary material for: Effects of COVID-19 lockdowns on unintended pregnancies among adolescent girls and young women in low- and middle-income countries: a scoping review
Source: Reprod Health. 2025 May 22;22:89. doi: 10.1186/s12978-025-02045-7 (PMC12096587; doi:10.1186/s12978-025-02045-7)
Supplement: Supplementary file 4 — Additional file 4. Table - Detailed overview of included publications. [file 12978_2025_2045_MOESM4_ESM.docx]

| **#** | **Author & publication year** | **Design** | **Country & *setting*** | **Study Population** | **Sample Size (n=)** | **Female** | **Classification of pregnancy** | **Outcomes of Interest** |
| --- | --- | --- | --- | --- | --- | --- | --- | --- |
| 1 | Adelekan et al; 2024 | Qualitative; FGD | Nigeria  *Urban and rural* | Married and unmarried adolescents & older women of reproductive age (15≥20 years) | 94 | 100% | Unplanned pregnancy | Increase in unplanned pregnancies resulting from COVID-19 lockdown |
| 2 | AfriChild; 2021 | Quantitative and qualitative; retrospective approach; phone survey; FGD^[[1]](#footnote-1)^, KII^[[2]](#footnote-2)^ & IDI^[[3]](#footnote-3)^; document review | Uganda  *Urban and rural* | IDIs with children (aged 10-17), KIIs with duty bearers at community and district level including parents, local leaders, Probation and Social Welfare Officers (PSWOs), Community Development Officers (CDOs), members of village health teams (VHTs), para-social workers among others | 1 800 phone survey respondents + participants in qualitative study | 60% | Teenage pregnancy | Increase in teenage pregnancies resulting from COVID-19 lockdown and school closures  8 teenage pregnancy cases in April 2019 vs 23 cases in September 2020 |
| 3 | Chimbindi et al; 2022 | Qualitative, IDI & FGD | South Africa  *Rural* | Teachers, peer-navigators (18-30 years) and learners (≥ 18 years) from 4 schools | 69 | 62.32% | Teenage pregnancy | Perceived increase in teenage & early pregnancies among learners; school drop out |
| 4 | Elsaid; 2022 | Quantitative, retrospective observational study | Egypt  *Urban and rural* | Women in a stable marital relationship (≥ 18 years) | 409 | 100% | Unwanted pregnancy | 45.5% participants reported pregnancies; 70% of pregnancies reported to have been unintended |

| **#** | **Author & publication year** | **Design** | **Country & *setting*** | **Study Population** | **Sample Size (n=)** | **Female** | **Classification of pregnancy** | **Outcomes of Interest** |
| --- | --- | --- | --- | --- | --- | --- | --- | --- |
| 5 | FAWE; 2021 | Quantitative and qualitative; cross-sectional survey and structured questionnaire; FGD | Uganda  *Urban and rural* | 3 258 girls aged 10-24; 3 136 boys; parents/guardians, teachers, and key informants | 6394 | 50.95% | Adolescent pregnancy | 22.5% increase in pregnancy among girls between March and June 2020; highest increase among girls aged 10-14 (366.5%) compared to girls aged 15-19 (25.5%) and young women aged 20-24 (21.1%) |
| 6 | Haddad et al; 2022 | Quantitative, cross-sectional | Lebanon  *Urban and rural* | married women between 18-51 | 369 | 100% | Unintended pregnancy assessed using binary question | 22% reported unwanted pregnancies |
| 7 | Khan et al; 2022 | Qualitative; semi-structured FGD & KII | Uganda  *Rural* | Youth 18–24-year-olds; mothers of youth; community leaders; Community Health Worker (CHWs) Coordinators, CHW supervisors, health providers, facility managers, district health managers, and district health officers | 94 | 18.09% | Unintended & teenage pregnancy | Perceived increase in youth pregnancy due to school closures |
| 8 | Mambo et al; 2022 | Quantitative, cross-sectional | Uganda  *Urban and rural* | Youth aged 18-30 years; 54.6% 18–24-year-olds | 733 | 43.60% | Unwanted pregnancies | Unwanted pregnancy one of the most common SRH problems reported during COVID-19 lockdown (32.4%) |

| **#** | **Author & publication year** | **Design** | **Country & *setting*** | **Study Population** | **Sample Size (n=)** | **Female** | **Classification of pregnancy** | **Outcomes of Interest** |
| --- | --- | --- | --- | --- | --- | --- | --- | --- |
| 9 | Musinguzi et al; 2022 | Quantitative, cross-sectional | Uganda  *Rural* | schoolgirls 13-19 years | 314 | 100% | Teenage pregnancy | 3 out of 10 teenage girls got pregnant during COVID-19; slight increase in teenage pregnancies |
| 10 | Mustafa; 2022 | Quantitative, prospective cross-sectional study | Pakistan  *Rural* | Women of reproductive age (18-45 years) | 350 | 100% | Unplanned pregnancy | 26.6% of women reported pregnancies; 80.6% out of those reported these to have been unplanned |
| 11 | Tenaw et al; 2022 | Quantitative, cross-sectional | Ethiopia  *Urban and rural* | Women attending antenatal care at public hospitals (15-49 years) | 421 | 100% | Unintended pregnancy - differentiate between mistimed and unwanted | 19.5% prevalence of unintended pregnancy; 50.6% mistimed and 49.4% unwanted; below 20-year-olds: 54.1% experienced unintended pregnancy |
| 12 | Women Deliver; 2022 | Combination of secondary data analysis and primary qualitative data collection methods | India, Kenya & Nigeria  *Urban and rural* | Key informants, youth advocates | 46 | - | Unintended and adolescent pregnancy | Perceived increase in unintended pregnancies as result of COVID-19 lockdown and reduced access to family planning |
| 13 | Zulaika et al; 2022 | Quantitative; nested longitudinal study | Kenya  *Rural* | Girls in their last 2 years of secondary school (mean age 17.2 years) | 910 | 100% | Adolescent pregnancy | School disruption leading to two times the risk of adolescent pregnancy in the COVID-19 cohort |

1. FGD – Focus Group Discussion [↑](#footnote-ref-1)
2. KII – Key Informant Interviews [↑](#footnote-ref-2)
3. IDI – In-depth interview [↑](#footnote-ref-3)
